# 7-Ketocholesterol Effects on Osteogenic Differentiation of Adipose Tissue-Derived Mesenchymal Stem Cells

**DOI:** 10.3390/ijms252111380

**Published:** 2024-10-23

**Authors:** Beatriz Araújo Oliveira, Débora Levy, Jessica Liliane Paz, Fabio Alessandro de Freitas, Cadiele Oliana Reichert, Alessandro Rodrigues, Sérgio Paulo Bydlowski

**Affiliations:** 1 Lipids, Oxidation, and Cell Biology Team, Laboratory of Immunology (LIM19), Heart Institute (InCor), Hospital das Clinicas HCFMUSP, Faculdade de Medicina, Universidade de Sao Paulo, Sao Paulo 05403-900, SP, Brazild.levy@hc.fm.usp.br (D.L.); fabio.alessandro@alumni.usp.br (F.A.d.F.); kadielli@hotmail.com (C.O.R.); 2 Department of Earth and Exact Sciences, Universidade Federal de Sao Paulo, Diadema 09972-270, SP, Brazil; alessandro.rodrigues@unifesp.br; 3 National Institute of Science and Technology for Regenerative Medicine (INCT Regenera), National Council for Scientific and Technological Development (CNPq), Rio de Janeiro 21941-902, RJ, Brazil

**Keywords:** oxysterol, 7-KC, mesenchymal stem cell, osteogenic differentiation, adipose tissue, Wnt/β-catenin

## Abstract

Some oxysterols were shown to promote osteogenic differentiation of mesenchymal stem cells (MSCs). Little is known about the effects of 7-ketocholesterol (7-KC) in this process. We describe its impact on human adipose tissue-derived MSC (ATMSC) osteogenic differentiation. ATMSCs were incubated with 7-KC in osteogenic or adipogenic media. Osteogenic and adipogenic differentiation was evaluated by Alizarin red and Oil Red O staining, respectively. Osteogenic (*ALPL, RUNX2, BGLAP*) and adipogenic markers (*PPARƔ, C/EBPα*) were determined by RT-PCR. Differentiation signaling pathways (SHh, Smo, Gli-3, β-catenin) were determined by indirect immunofluorescence. ATMSCs treated with 7-KC in osteogenic media stained positively for Alizarin Red. 7-KC in adipogenic media decreased the number of adipocytes. 7-KC increased *ALPL* and *RUNX2* but not *BGLAP* expressions. 7-KC decreased expression of *PPARƔ* and *C/EBPα*, did not change SHh, Smo, and Gli-3 expression, and increased the expression of β-catenin. In conclusion, 7-KC favors osteogenic differentiation of ATMSCs through the expression of early osteogenic genes (matrix maturation phase) by activating the Wnt/β-catenin signaling pathway, while inhibiting adipogenic differentiation. This knowledge can be potentially useful in regenerative medicine, in treatments for bone diseases.

## 1. Introduction

Cell differentiation is a process in which a less specialized cell becomes a more specialized cell type. Mesenchymal stem cells (MSCs) are defined as undifferentiated cells with the capability to differentiate into several cell types [1]. Several processes can influence stem cell differentiation such as gene regulation, cell–cell contact [2] mitochondrial activity [3], the mechanical properties of the microenvironment [4], and activation or silencing of specific transcription programs in response to environmental stimuli, among others [5]. The multipotent differentiation ability of mesenchymal stem cells has made them a subject of great interest due to their potential therapeutic clinical applications [6]. In fact, this property is a promising tool to improve strategies in regenerative medicine and tissue engineering in several key clinical situations such as immunosuppression, neurodegenerative disorders, myocardial infarction, and wound healing.

Osteogenesis and adipogenesis are among the cell differentiation process of MSCs. Differentiation into osteogenic tissue can be particularly beneficial in treating bone defects, fractures, osteoporosis, and conditions like osteoarthritis. Differentiation into adipose tissue can be used in reconstructive surgery, such as breast reconstruction, or in cosmetic procedures where fat tissue is needed.

In osteogenesis, a cell derived from bone marrow MSCs, the osteoblast, is involved in the synthesis and mineralization of bone formation [7]. Adipogenesis corresponds to the differentiation of preadipocytes into adipocytes, which are cells specialized in lipid accumulation. Osteogenesis occurs under the influence of several molecules, such as ALPL, SHh, and RUNX2, while adipogenesis occurs under the action of several transcription factors, such as PPARƔ and various members of the C/EBP family [8,9].

Cholesterol is a structural component of cell membranes [10,11]. It acts on the regulation of several biological processes [12] and can be easily oxidized by enzymatic or non-enzymatic processes into products known as oxysterols [10,13]. Oxysterols comprise a large family of 27-carbon molecules found in tissues at very low concentration that act in a number of physiological processes such as platelet aggregation, cholesterol homeostasis regulation, and cell death, including that of stem cells [6,14,15].

7-KC has been described as one of the most important oxysterols, mainly generated by the autoxidation of cholesterol [16,17,18]. It is known to be involved in several physiological and pathophysiological processes, such as oxidative stress, monocyte differentiation, cardiovascular diseases and atherosclerosis, Alzheimer’s disease, colon carcinoma, breast cancer, age-related macular degeneration and cataracts [16,18,19], among others [20,21]

7-KC participates in the promotion of cell death, including the induction of a particular type known as oxiapoptophagy, in which a simultaneous combination of OXIdative stress, APOPTOsis, and autoPHAGY is present [22,23], a concept first introduced by Lizard et al. [24,25]. Oxidative stress is associated with the overproduction of ROS, increased antioxidant enzyme activities, and lipid peroxidation, while apoptosis is associated with activation of the mitochondrial pathway, loss of mitochondrial membrane potential, caspase-3 activation, and autophagy with autophagic vacuoles, among other features.

Although very little studied, oxysterols that interfere with MSC differentiation can be potentially useful to improve differentiation strategies. Currently, 22(*R*)- or 22(*S*)- and 20(*S*)-hydroxycholesterol are the main oxysterols studied in MSC osteogenic differentiation. 20(*S*)-hydroxycholesterol has been described to increase osteogenic activity in vitro and in vivo, being able to induce bone formation from MSCs derived from bone marrow and also from embryonic stem cells. The joint use of the oxysterols 22(*R*)- or 22(*S*)- and 20(*S*)-hydroxycholesterol enhances the osteogenic activity through the activation of the Hh (Hedgehog) signaling pathway indirectly, through the activation of Smo [6,26,27,28,29,30].

Despite this knowledge, little is known regarding the effects of 7-KC in this process [6,29]. After 24 h of treatment, high concentrations of 7-KC promoted mitochondrial hyperpolarization, increased apoptosis and changed actin organization in adipose tissue-derived mesenchymal stem cells (ATMSCs) [10,15]. In this regard, few studies with ATMSCs from obese patients have shown that 7-KC, besides decreasing cell viability of adipose precursor cells, prevents adipogenic differentiation through the activation of Wnt and MAPK signaling pathways, which are also involved in osteogenic differentiation [15,30,31].

Here, we analyzed the effects of 7-KC on the osteogenic and adipogenic differentiation of MSCs derived from human adipose tissue using several osteogenic and adipogenic markers. In addition, the effects of 7-KC on major differentiation signaling pathways, including the Wnt/β-catenin and Sonic Hedgehog pathways, were evaluated.

## 2. Results

### 2.1. Induction of Osteogenic Differentiation

**Figure 1:**
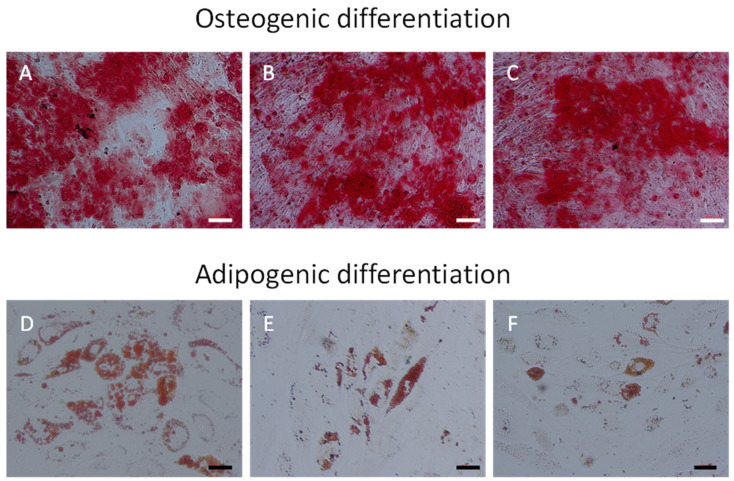
Osteogenic and adipogenic differentiation of ATMSCs. Effect of 7-KC. Osteogenic differentiation: (**A**): Control, ATMSCs cultivated for 21 days with osteogenic specific medium alone. (**B**,**C**): Osteogenic medium with 3 and 5 µM 7-KC, respectively. Staining with Alizarin Red. Bar scale: 12.5 µm. Adipogenic differentiation: (**D**): Control, ATMSCs cultivated for 14 days with adipogenic specific medium. (**E**,**F**): Adipogenic medium with 3 and 5 µM 7-KC M, respectively. Staining with Oil Red O. White bar scale: 200 µm, black bar scale: 50 µm.

Incubation of ATMSCs for 21 days with osteogenic differentiation medium led to osteogenic differentiation as evaluated by calcium deposition, detected using Alizarin Red (Figure 1A). 7-KC alone (3 to 15 µM), without osteogenic differentiation medium, was not able to induce osteogenic differentiation, as evaluated by calcium deposition. Higher concentrations (>15 µM) of 7-KC were cytotoxic, independently of the presence of osteogenic medium. ATMSCs treated with osteogenic specific medium and 7-KC at concentrations of 3 to 15 µM stained positively for Alizarin Red (Figure 1B,C and Figure S1, Supplementary Materials).

### 2.2. Induction of Adipogenic Differentiation

Incubation of ATMSCs for 14 days with adipogenic differentiation medium led to adipogenic differentiation as evaluated by Oil red O staining (Figure 1). 7-KC at concentrations of 10–30 µM decreased cell viability, independently on the presence of adipogenic medium. ATMSCs incubated with lower concentrations of 7-KC alone did not lead to adipogenic differentiation. The same concentrations of 7-KC (3 and 5 µM) incubated with adipogenic medium led to a decrease in the number of adipocytes, in a concentration-dependent manner (Figure 1E,F).

### 2.3. ATMSC Gene Expression by RT-PCR

#### 2.3.1. Expression of *RUNX2*

**Figure 2:**
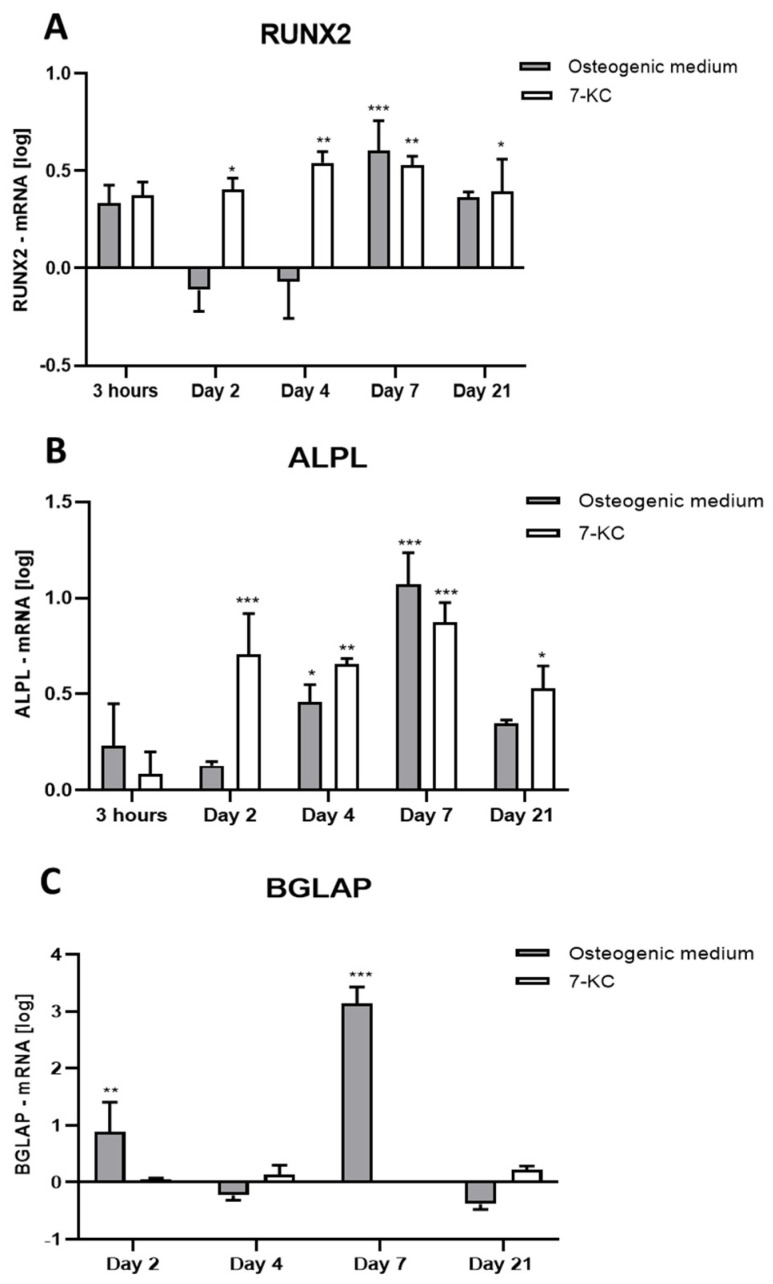
Expression of osteogenic differentiation markers in ATMSCs as measured by RT-PCR. ATMSCs were incubated with osteogenic specific medium with or without 10 µM 7-KC for different periods of time. (**A**): *RUNX2*. (**B**): *ALPL*. (**C**): *BGLAP*. Data are mean ± SD from three independent experiments in duplicate. Results are shown in log scale. * *p* < 0.05; ** *p* < 0.01; *** *p* < 0.0001.

The expression of *RUNX2* was measured after treatment with osteogenic differentiation medium, with or without 7-KC (10 µM) in different time periods (Figure 2A). Incubation of ATMSCs with specific osteogenic medium alone led to an increase in *RUNX2* expression only at the 7th day; no changes were observed in other periods. The addition of 7-KC increased *RUNX2* expression on the 2nd day, remaining highly expressed until the 21st day.

#### 2.3.2. Expression of *ALPL*

The expression of *ALPL* was measured after treatment with osteogenic differentiation medium, with or without 7-KC (10 µM), in different time periods (Figure 2B). Osteogenic medium alone increased the expression of *ALPL* at the 4th and 7th days, returning to basal levels at day 21. Again, the addition of 7-KC increased *ALPL* expression on the 2nd day, remaining highly expressed until the 21st day.

#### 2.3.3. Expression of *BGLAP*

The expression of *BGLAP* was measured after treatment with osteogenic differentiation medium, with or without 7-KC (10 µM), in different time periods (Figure 2C). After 3 h incubation, none of the treatments changed *BGLAP* expression values. Unexpectedly, specific osteogenic medium increased the expression of *BGLAP* only at the 2nd and 7th days of differentiation induction. Treatment with 7-KC did not change *BGLAP* expression in any of the evaluated periods of time.

#### 2.3.4. Expression of *PPARƔ*

**Figure 3:**
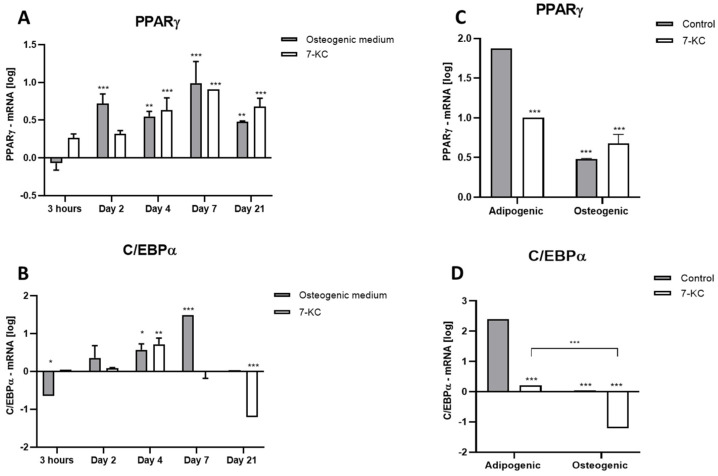
Expression of adipogenic differentiation markers in ATMSCs as measured by RT-PCR. ATMSCs were incubated with osteogenic specific medium with or without 10 µM 7-KC for different periods of time. (**A**): *PPARƔ* expression. (**B**): *C/EBPα* expression. ATMSCs were incubated with osteogenic or adipogenic specific medium with or without 10 µM 7-KC for 21 days. (**C**): *PPARƔ* expression. (**D**): *C/EBPα* expression. Data are mean ± SD from three independent experiments in duplicate. Results are expressed in log scale. * *p* < 0.05; ** *p* < 0.01; *** *p* < 0.0001.

We have also determined the effect of osteogenesis on the expression of the adipogenic markers PPARƔ and C/EBPα. Figure 3A shows PPARƔ expression as affected by 7-KC at different time periods. Incubation with specific osteogenic medium alone increased PPARƔ expression at the 2nd day. PPARƔ remained highly expressed until the 21st day of treatment. 7-KC led to higher expression levels of PPARƔ from the 4th day until the 21st of incubation.

The unexpected increase in *PPARƔ* expression promoted by osteogenic medium and 7-KC led us to compare these data with those obtained by the incubation of ATMSCs with adipogenic medium after 21 days, with or without 7-KC (Figure 3B). We have found that both media (adipogenic and osteogenic) and 7-KC led to an increase in *PPARƔ* expression. Nevertheless, results using osteogenic specific medium were much lower compared with those obtained by incubation with adipogenic media. Moreover, 7-KC decreased the expression of *PPARƔ* during adipogenic differentiation when compared to cells treated with specific adipogenic medium alone.

#### 2.3.5. Expression of *C/EBPα*

ATMSCs treated for 3 h with osteogenic specific medium expressed lower levels of *C/EBPα* expression, which increased from the 2nd to the 7th days of treatment; no expression was seen on the 21st day (Figure 3C). In cells treated with 7-KC, *C/EBPα* expression increased at the 4th day and strongly decreased at the 21st day. In the other measured periods of time, 7-KC did not alter the *C/EBPα* expression.

Similarly to *PPARƔ*, we have compared the osteogenic medium incubation data with those obtained by the incubation of ATMSCs with adipogenic medium on day 21, with or without 7-KC (Figure 3D). Again, *C/EBPα* expression was higher in cells treated with specific adipogenic medium than in cells stimulated to osteogenic differentiation. 7-KC decreased *C/EBPα* expression. This effect was strongly enhanced when cells were incubated with osteogenic medium compared with those incubated with adipogenic medium.

### 2.4. Differentiation Signaling Pathways

Some differentiation signaling pathways were evaluated by the intensity of fluorescence after 7 days of incubation with 7-KC, osteogenic or adipogenic media.

**Figure 4:**
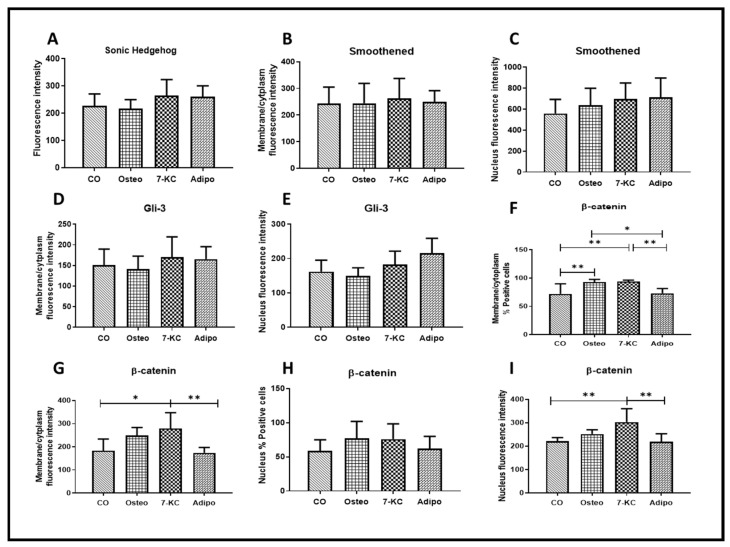
Osteogenic signaling pathways measured by immunofluorescence. Effect of 7-KC and osteogenic and adipogenic media. (**A**): Mean fluorescence intensity of SHh expression. (**B**,**C**): Mean fluorescence intensity of Smo expression in membrane/cytoplasm and nucleus, respectively. (**D**,**E**): Mean fluorescence intensity of Gli-3 expression in membrane/cytoplasm and nucleus, respectively. (**F**): Percentage of cells expressing β-catenin in membrane/cytoplasm. (**G**): Mean fluorescence intensity of β-catenin expression in membrane/cytoplasm. (**H**): Percentage of cells expressing β-catenin in the nucleus. (**I**): Mean fluorescence intensity of β-catenin expression in the nucleus. Data are mean ± SD from three independent experiments in duplicate. * *p* < 0.05; ** *p* < 0.01.

7-KC and media did not change SHh expression (Figure 4A). The expression of Smo and Gli-3 in the nucleus and in membrane/cytoplasm also did not change with different treatments (Figure 4B–E).

7-KC and osteogenic medium both increased the percentage of β-catenin-positive cells in the membrane/cytoplasm (Figure 4F). Only 7-KC increased β-catenin in the membrane/cytoplasm, as evaluated by the intensity of fluorescence (Figure 4G). In the nucleus, β-catenin-positive cells did not change with any treatment (Figure 4H). 7-KC increased β-catenin in the membrane/cytoplasm as evaluated by the intensity of fluorescence (Figure 4H). Osteogenic and adipogenic media had no effect.

## 3. Discussion

Studies on the effect of 7-KC on osteogenic and adipogenic differentiation of MSCs are lacking. Here, we evaluated the capacity of 7-KC in promoting these differentiations in adipose tissue-derived MSCs. We have shown that ATMSCs treated with osteogenic specific medium and 7-KC at concentrations of 3 to 15 µM stained positively for Alizarin Red. However, 7-KC had no effect in the absence of osteogenic media. Treatment with adipogenic media and 7-KC (3 and 5 µM) led to a decrease in the number of adipocytes as determined by Oil red O. Again, no effect was seen in the absence of adipogenic media.

It has been described that three developmental phases are present during osteogenic differentiation in bone marrow-derived MSCs: proliferation from the 0th to 4th day; matrix maturation from the 4th to 14th day, and mineralization from the 14th to 21st day [32].

An increase in the expression of early osteogenic markers such as *ALPL* and *RUNX2* has been observed from day 4. Interestingly, our results showed that 7-KC increased the expression of *ALPL* and *RUNX2* on the 2nd day, suggesting an earlier onset of matrix maturation in these cells. Whether time development of those phases is different depending on the origin of MSCs is not known.

*BGLAP* is a late differentiation marker gene that encodes osteocalcin [33,34]. After commitment to osteoblastic lineage, cells express bone matrix protein genes at different levels according to their maturational stage [35]. *BGLAP* expression strongly increased on the 7th day as an effect of incubation with osteogenic media alone. However, the addition of 7-KC did not change *BGLAP* expression, thus suggesting that they were not able to induce ATMSC matrix mineralization and differentiation into mature osteoblasts, even in the presence of osteogenic medium, at least in the observed period.

*ALPL* expression was described to increase during osteogenic differentiation followed by a decrease in the mineralization phase; however, this increase was similar to that observed during adipogenic differentiation [36]. Here, we have shown that during osteogenic induction there is, in addition to the increase in osteogenic markers, an increase in the expression of adipogenic markers, such as *PPARƔ* and *C/EBPα*. However, the expression of these genes was much lower than that observed during adipogenic differentiation. Moreover, we have found that 7-KC increased the expression of osteogenic genes while inhibiting the expression of adipogenic genes such as *PPARƔ* and *C/EBPα*, even under adipogenic differentiation stimuli, supporting its anti-adipogenic action.

Wnt comprises a family of molecules involved in mammalian cell differentiation, proliferation, and migration. In the canonical cellular signaling by Wnt, β-catenin is stabilized and translocated to the nucleus. In the absence of Wnt, β-catenin is destroyed by proteasomes [37,38,39].

Another molecule described to be involved in osteogenic signaling is SHh, a member of a Hh family [40]. First, an active Hh ligand is generated, which relieves tonic inhibition of the receptor Smo. Subsequently, Smo activates SHh signaling and then the translocation of the Gli transcription factor into the nucleus [41,42]. Both signaling pathways, Wnt/β-catenin and SHh, are important during osteogenesis through the induction of the expression of osteogenic markers such as *ALPL* and *RUNX2* [29,43,44].

Here, we described that SHh, Smo, and Gli-3 did not change with any treatment, despite the increase in the expression of β-catenin promoted by 7-KC after seven days of treatment. A lack of response of SHh to 7-KC has been already described in a study with human breast cancer cells [20]. However, studies with MSCs derived from the bone marrow of patients with acute myeloid leukemia described a decrease in SHh protein expression promoted by 7-KC without changes in Smo expression [18]. Other studies have shown that SHh and Smo are involved in osteogenic differentiation and are highly expressed by the action of oxysterols [28,29]. However, our results demonstrated that osteogenic differentiation occurs by the action of 7-KC without the interference of the SHh signaling pathway. Further studies are needed to better understand how SHh pathways are affected by oxysterols in terms of the type and origin of cells and influence of the concentration and time of treatment.

## 4. Materials and Methods

### 4.1. Reagents, Kits and Plastics

Reagents and media: fetal bovine serum (FBS) (Vitrocell, Waldkirch, Germany); solution trypsin-EDTA (Gibco, Waltham, MA, USA); collagenase type IV (Molecular Probes, Eugene, OR, USA); 2-propanol (Merck, Rahway NJ, USA); Trizol (Invitrogen, Carlsbad, CA, USA). All other products were from Sigma Aldrich, St. Louis, MO, USA: Dulbecco’s Modified Eagle Medium (DMEM); streptomycin; penicillin; dexamethasone; β-glicerophosphate disodium salt hydrate; L-ascorbic acid 2-phosphate sesquimagnesium salt; indomethacin; insulin; 3-isobutyl-1-methylxanthine; paraformaldehyde; Alizarin red; Oil Red O; cholesterol; Triton X-100; BSA (bovine serum albumin).

Culture flasks and well plates were from Santa Cruz Biotechnology, Dallas, TX, USA. The 96-well Black Flat Bottom Polystyrene Microplates were from Corning, Somerville, MA, USA.

Antibody anti-β-catenin (AB16051) and anti-Sonic Hedgehog (ab53281) were from Abcam, Cambridge, UK. Smoothened antibody (NBP2-24543) and GLI-3 antibody (H00002737-M01) were acquired from Novus Biologicals, Centennial, CO, USA. AlexaFluor 488 (A-11008) and anti-mouse R-phycoerynthrin (P852) were from Molecular Probes, Eugene, OR, USA.

RQ1 RNase-Free DNase was acquired from Promega—Madison, WI, USA. cDNA synthesis kit was from Applied Biosystems, Whaltam, MA, USA. GAPDH (402869) and TaqMan Gene Expression Master Mix were from Thermo Fisher, Waltham, MA, USA.

7-ketocholesterol was synthetized from cholesterol as previously described [18,45,46]. The purity of oxysterols was determined to be ~98% by GS/MS. The stock solution was prepared at a concentration of 10 mM in absolute ethanol. Six concentrations (3-30 µM) were selected based on IC50 results (59.54 µM 7-KC) described by Silva et al., 2016 [10].

The stock solution of Oil Red O was prepared by dissolving 0.25 g of the dye in 50 mL 2-propanol, followed by filtration. At the time of staining, the working solution was prepared mixing 30 mL of stock solution with 20 mL Milli-Q water followed by filtration in Whatman filter paper.

### 4.2. ATMSC Isolation and Characterization

The Ethical Committee of the Institution approved the protocol for this study and patients provided written informed consent. The adipose tissue was obtained from three healthy women (age between 20 and 45 years old) that underwent abdominal plastic surgery due to aesthetic reasons. From each patient, 30 mL of fatty material was collected in a sterile flask and processed as described [6]. Briefly, the tissue was dissociated with 30 mg of collagenase type IV diluted in 30 mL of DMEM low glucose for 45 min, and then centrifuged to isolate the cells. Medium consisting of 12 mL of DMEM supplemented with 20% heat-inactivated FBS and 1% streptomycin-100 µg/mL and penicillin-100 UI/mL (200 µL final volume) were added to the cell pellet. After transferring to 75 cm^2^ culture flasks, cells were incubated at 37 °C in 5% CO_2_ atmosphere. Before reaching confluence, cells were detached using a trypsin-EDTA solution and seeded at a density of 5 × 10^3^ cells/cm^2^. Cells were used for experiments at the 5th passage. ATMSC characterization was performed according to methods preconized by The International Society for Cellular Therapy [47], including markers measured by flow cytometry, as previously described [10,15].

### 4.3. Osteogenic Differentiation

ATMSCs from each donor were plated at a density of 1 × 10^4^ cells/cm^2^ in duplicate in 96-well tissue culture plates or in 6-well tissue culture plates, for RNA extraction, and incubated in DMEM low glucose medium containing 20% FBS and 1% antibiotics at 37 °C in 5% CO_2_ atmosphere. Two days after seeding, cells were kept on two different media: 1. DMEM low glucose supplemented with 20% of FBS (basal medium), or 2. an osteogenic medium consisting of DMEM low glucose with 20% FBS, 0.1 µM dexamethasone, 10 mM β-glycerophosphate disodium salt hydrate, and 50 µM L-ascorbic acid 2-phosphate sesquimagnesium salt. These are the most common components used for the induction of osteogenic differentiation in vitro. The combination of these components activates specific signaling pathways and gene expression such as Wnt/β-catenin [8,14,48].

7-KC was added to the media in different concentrations (0, 3, 5, 10, 15, 20, or 30 µM, 50 µL final volume). Treatment lasted up to 21 days and the medium was replaced every 3 days. Following this, medium was removed and cells were fixed in 4% paraformaldehyde for 2 h at 4 °C. To evaluate the osteogenic differentiation, cells were washed twice with Dulbecco’s Phosphate Buffered Saline (DPBS) and then stained with Alizarin red (1 g of dye in 50 mL Milli-Q water, pH 4.2) for 3 min at room temperature and washed 3 times with DPBS.

### 4.4. Adipogenic Differentiation

Two days after seeding, cells were cultured in two different media: 1. DMEM high glucose with 2% FBS (basal medium), and 2. adipogenic medium consisting of DMEM high glucose with 2% FBS, 1 µM dexamethasone, 2 µM indomethacin, 1 µg/mL insulin, and 0.5 mM 3-isobutyl-1-methylxanthine (IBMX) [8,49]. From the 10th day on, the concentration of insulin in the adipogenic specific medium was increased to 10 µg/mL. 7-KC was added to the media in different concentrations (0, 3, 5, 10, 15, 20, or 30 µM, 50 µL final volume). Treatment lasted up to 21 days and the medium was replaced every 3 days. At the end of the treatment, adipogenic differentiation was evaluated by the accumulation of neutral cytoplasmic lipid vacuoles (Oil Red O staining). Briefly, medium was removed and cells were fixed with 4% paraformaldehyde for 2 h at 4 °C. After washing twice with DPBS, cells were incubated with Oil Red O solution for 20 min at room temperature followed by washing with Milli-Q water twice to remove excess stain. The culture plates were visualized using phase-contrast microscopy (20×), Axiovert 5 (Carl Zeiss microscopy, Inzelmann, Germany).

### 4.5. RNA Extraction

Total RNA was extracted from cells with Trizol reagent according to the manufacturer’s instructions. This process was carried out at 5 different times during the differentiation period (3 h, 2 days, 4 days, 7 days, and 21 days). The purity of RNA was determined by the absorbance ratio (260 to 280 nm). RNA (1 µg) was incubated with RQ1 RNase-Free DNase as described by the manufacturer. cDNA synthesis was performed as described by the manufacturer. A thermal cycler XP Cycler (Bioer Technology—Hangzhou, China) was used for incubation.

### 4.6. Expression of Genes Related to AMSC Differentiation—RT-PCR

Expression of several different genes related to ATMSC differentiation was evaluated by Real-Time PCR:

- *RUNX2*, a member of a transcription factor family required for MSC differentiation [50,51] is also an osteogenic marker; its action occurs through the binding with *Cbfβ* (Core Binding Factor β) leading to a decrease in adipogenic markers [50,52].
- *ALPL*, which encodes a protein used as a marker for osteogenic differentiation [32,53]; the *ALPL* gene expression is considered an early marker of osteogenic differentiation [32,53]; *ALPL* is involved in MSC fate determination and bone aging through the regulation of release and hydrolysis of ATP and AMPKα pathway [53].
- *BGLAP*, which encodes osteocalcin, is strongly expressed in mature osteoblasts [35]; it is used as a serum marker of bone formation and is believed to enhance bone matrix mineralization [33].
- *C/EBPα*, an adipogenic marker, which encodes a transcription factor commonly expressed in adipose tissue, is involved in terminal differentiation of adipocytes [9].
- *PPARƔ*, also an adipogenic marker, is a member of the Peroxisome Proliferator family that, through heterodimerization with RXR (Retinoid X Receptor), regulates genes of cellular differentiation [6]; it is also described to be involved in terminal differentiation of adipocytes [8].

The expression of mRNA was normalized to endogenous glyceraldehyde-3-phosphate dehydrogenase (GAPDH) using the comparative cycle threshold (CT); duplicates did not exceed the 0.5 CT value. The primers for *RUNX2* (Hs.PT.56a.19568141), *ALPL* (Hs.PT.56a.40555206), *BGLAP* (Hs.PT.56a.39318706.g), *C/EBPα* (Hs.PT.58.4022335.g) and *PPARƔ* (Hs.PT.58.25464465) were obtained from Integrated DNA Technologies (Coralville, IA, USA) with pre-designed tests for hydrolyzable probes (Table 1). TaqMan Gene Expression Master Mix was used as described by the manufacturer. Tests were performed using 2^−ΔΔCT^ methodology [54]. Data are presented as log of the medium/basal medium ratio differentiation.

### 4.7. Differentiation Signaling Pathways Measured by Indirect Immunofluorescence

Cells were plated at density of 1 × 10^3^ cells/cm^2^ in 96-well Black Flat Bottom Polystyrene Microplates and treated with osteogenic specific medium with 7-KC (10 µM). Control cells were cultivated in DMEM low glucose with 20% FBS or osteogenic medium or adipogenic medium. After 7 days, cells were fixed with 4% paraformaldehyde for 2 h at 4 °C and then washed twice with DPBS. Cells were permeabilized with 0.1% Triton X-100 solution for 15 min at 4 °C, washed twice with DPBS and blocked with 5% BSA (bovine serum albumin) at room temperature for 40 min. Cells were incubated overnight with various antibodies: anti-β-catenin (1:500 dilution), anti-Smoothened antibody (1:100 dilution), anti-Sonic Hedgehog (1:400 dilution), or anti-GLI-3 antibody (1:100 dilution). Subsequently, cells were incubated for an additional 1 h with anti-rabbit secondary antibody, AlexaFluor 488 (1:1000 dilution) or anti-mouse R-phycoerynthrin (1:1000 dilution). The fluorescence intensities were determined with ImageXpress Micro High Content Screening System (Molecular Devices, Eugene, OR, USA). Five fields per well and two wells per treatment were acquired. Data were analyzed using the Cells Scoring MetaXpress software (v5, Molecular Devices, Eugene, OR, USA).

### 4.8. Statistical Analysis

Results are expressed as mean ± SEM from at least three independent experiments. Means were compared using One- and Two-way ANOVA followed by Dunnet’s multiple comparisons post-test and Bonferroni post-test on GraphPad Prism (GradPad Software v8). *p*-values ˂ 0.05 were considered significant.

## 5. Conclusions

This study brings new information on the biological activity of 7KC and shows differentiating properties of this oxysterol on mesenchymal stem cells. Thus, 7-KC favors osteogenic differentiation of adipose tissue-derived mesenchymal stem cells through the earlier expression of early osteogenic genes (matrix maturation phase) by the activation of the Wnt/β-catenin signaling pathway. However, 7-KC had no effect on the expression of late markers of osteogenic differentiation, suggesting that it does not induce matrix mineralization. Furthermore, it promotes the inhibition of adipogenic differentiation of these cells. Noteworthy, 7-KC was unable to increase the expression of components of the SHh signaling pathway. This knowledge will be useful in regenerative medicine, especially in potential treatments for bone diseases.

## Figures and Tables

**Table 1:** Primers for gene expression measured by RT-PCR.

Gene	SEQ REF	Assay ID (IDT)
*RUNX2*	NM_001024630(3)	Hs.PT.56a.19568141
*ALPL*	NM_001127501(3)	Hs.PT.56a.40555206
*BGLAP* (Osteocalcin)	NM_199173(1)	Hs.PT.56a.39318706.g
*CEBPα*	NM_004364(1)	Hs.PT.58.4022335.g
*PPAR* *Ɣ*	NM_005037(4)	Hs.PT.58.25464465

## Data Availability

The data presented in this study are available on request from the corresponding author.

## Supplementary Materials

The following supporting information can be downloaded at: https://www.mdpi.com/article/10.3390/ijms252111380/s1.
