# Supplementary material for: 7-Ketocholesterol Effects on Osteogenic Differentiation of Adipose Tissue-Derived Mesenchymal Stem Cells
Source: Int J Mol Sci. 2024 Oct 23;25(21):11380. doi: 10.3390/ijms252111380 (PMC11545361; doi:10.3390/ijms252111380)
Supplement: Supplementary file 1 [file ijms-25-11380-s001.zip › ijms-3231318-supplementary.pdf]

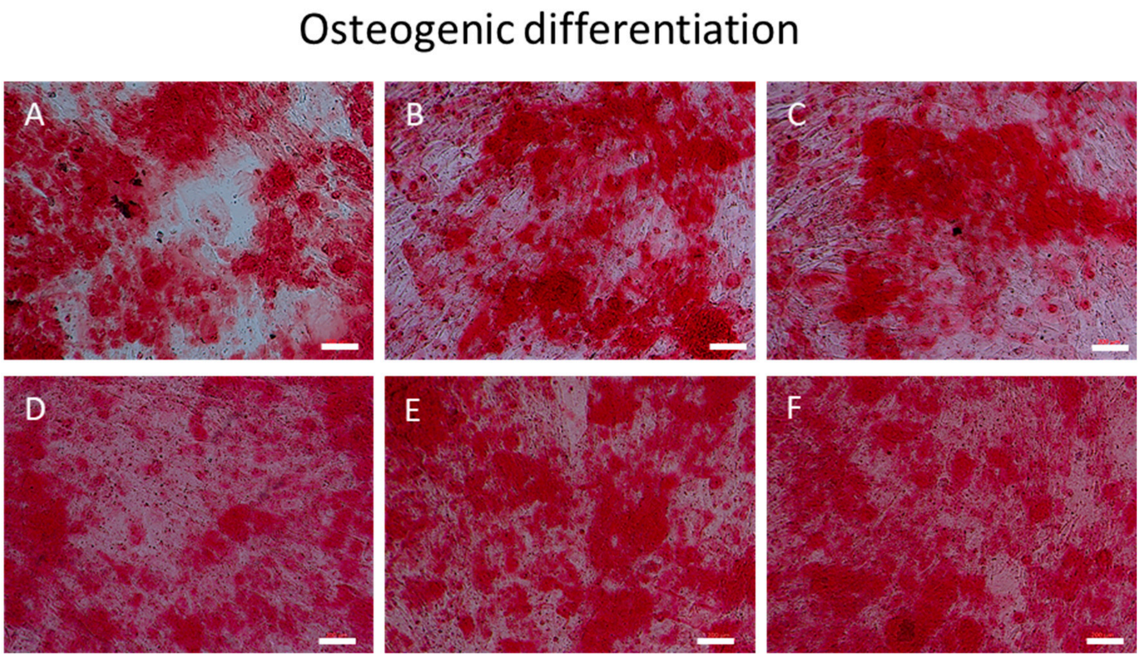

Figure S1. ATMSCs treated for 21 days with osteogenic specific medium and 7-KC in different concentrations. Cells were stained positively for Alizarin Red at concentrations of 3 to 20 μM. 7-KC was cytotoxic at 30 μM. **A:** Control, osteogenic specific medium. **B**: 7-KC 3 μM. **C**: 7-KC 5 μM. **D**: 7-KC 10 μM. **E**: 7-KC 15 μM. **F**: 7-KC 20 μM.
